# Crystal Structures Reveal that the Reaction Mechanism of Imidazoleglycerol-Phosphate Dehydratase Is Controlled by Switching Mn(II) Coordination

**DOI:** 10.1016/j.str.2015.05.012

**Published:** 2015-07-07

**Authors:** Claudine Bisson, K. Linda Britton, Svetlana E. Sedelnikova, H. Fiona Rodgers, Thomas C. Eadsforth, Russell C. Viner, Tim R. Hawkes, Patrick J. Baker, David W. Rice

**Affiliations:** 1 Department of Molecular Biology and Biotechnology, Krebs Institute for Biomolecular Research, University of Sheffield, Firth Court, Western Bank, Sheffield S10 2TN, UK; 2 Syngenta, Jealott's Hill International Research Station, Bracknell RG42 6EY, UK

## Abstract

Imidazoleglycerol-phosphate dehydratase (IGPD) catalyzes the Mn(II)-dependent dehydration of imidazoleglycerol phosphate (IGP) to 3-(1H-imidazol-4-yl)-2-oxopropyl dihydrogen phosphate during biosynthesis of histidine. As part of a program of herbicide design, we have determined a series of high-resolution crystal structures of an inactive mutant of IGPD2 from *Arabidopsis thaliana* in complex with IGP. The structures represent snapshots of the enzyme trapped at different stages of the catalytic cycle and show how substrate binding triggers a switch in the coordination state of an active site Mn(II) between six- and five-coordinate species. This switch is critical to prime the active site for catalysis, by facilitating the formation of a high-energy imidazolate intermediate. This work not only provides evidence for the molecular processes that dominate catalysis in IGPD, but also describes how the manipulation of metal coordination can be linked to discrete steps in catalysis, demonstrating one way that metalloenzymes exploit the unique properties of metal ions to diversify their chemistry.

## Highlights

• IGPD forms open and closed complexes with IGP bound in two distinct conformations
• Mn(II) controls catalysis by switching between 6- and 5-coordination states
• A ligand-depleted 5-coordinate Mn(II) aids formation of the imidazolate intermediate
• Enzyme and substrate conformational changes are required for product formation

## Graphical Abstract

**Figure:**
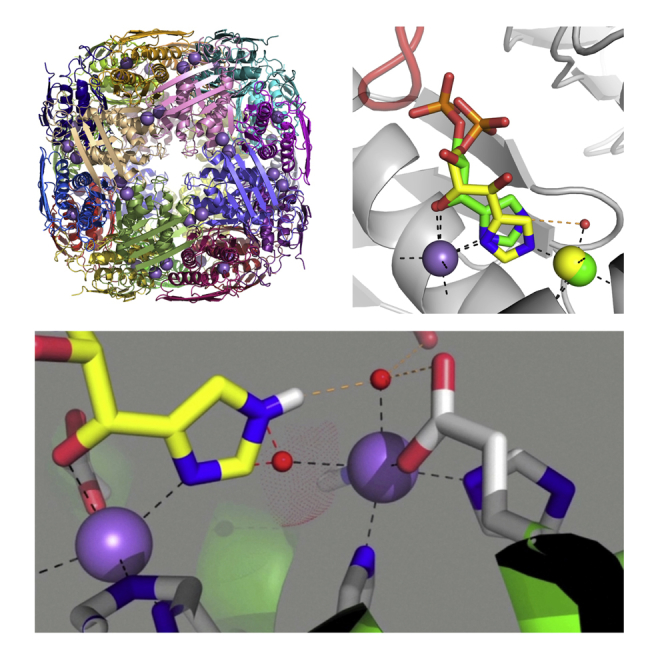

## Teaser

Bisson et al. determined structures that describe the reaction mechanism of IGPD, showing how the enzyme harnesses substrate binding energy to generate a ligand-depleted Mn(II) at the catalytic center. Conformational changes in the enzyme-substrate complex and switches in coordination chemistry control successive steps in catalysis.

## Introduction

Metalloenzymes account for more than half of known enzymes (Foster et al., 2014) and are fundamental in facilitating many of the core metabolic processes that are essential for life. The chemical diversity and functionality of metalloenzymes is dependent upon their ability to utilize the unique chemistry of metal ions to facilitate specific reactions (Finkelstein, 2009; Frausto da Silva and Williams, 2001; Karlin, 1993). Numerous biophysical, biochemical, and structural studies have provided insights into how metal ions promote catalysis, highlighting their involvement in bond polarization and stabilization of transition states and intermediates (Andreini et al., 2008). Moreover, in the case of certain redox enzymes, for example manganese superoxide dismutase (Abreu and Cabelli, 2010) and oxalate decarboxylase (Tabares et al., 2009), changes in the oxidation state of the metal ions have been associated with variations in ligand coordination geometry during the reaction (Andreini et al., 2008). However, there is still much to be learned about the exact roles that different metal ions play in driving enzyme catalysis, specifically how the exquisite specificity with which they are selected is matched to the chemistry in which they are involved.

**Figure 1:**
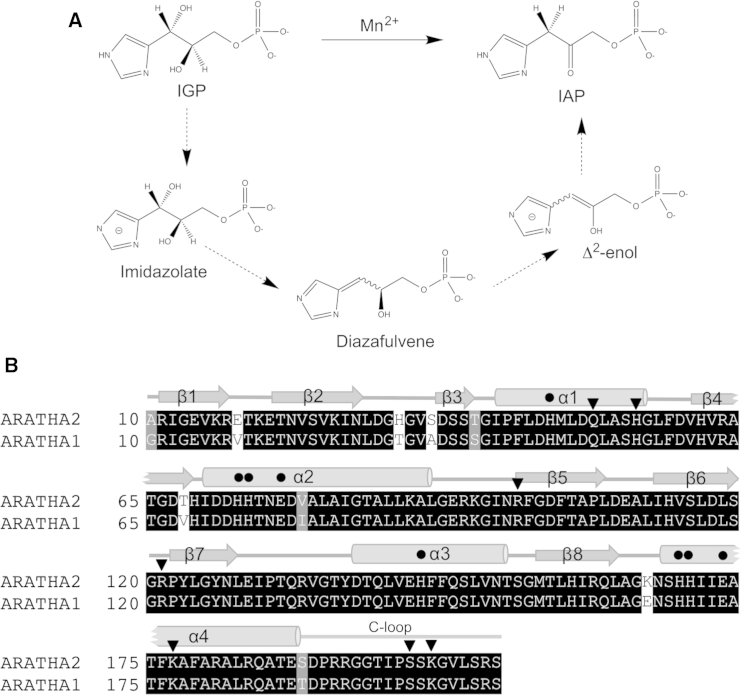
IGPD Catalyzes the Mn(II)-Dependent Dehydration of IGP to IAP (A) Schematic drawing representing an overview of the reaction mechanism of IGPD, highlighting the key intermediates. Figure generated using ChemDraw. (B) Structure-based sequence alignment showing equivalent residues in *A. thaliana* IGPD2 (ARATHA2) and IGPD1 (ARATHA1). Black and gray shading indicates identical or similar residues, respectively. Secondary structure elements are numbered and represented as gray arrows (beta strands), tubes (helices), and lines (loop regions) above the sequence. The C loop is labeled and shown as a gray line. Black dots indicate residues that are involved in metal ion binding. Residues implicated in recognition of the substrate-phosphate are marked with a black triangle. Numbering is based on the convention adopted in the structure of IGPD1 (PDB: 2F1D) (Glynn et al., 2005). Sequences were retrieved from UniProt, the alignment was produced using Tcoffee, and the figure was drawn using boxshade. See also Figure S1.

The metalloenzyme imidazoleglycerol-phosphate dehydratase (IGPD) (EC 4.2.1.19) has been identified as a potential herbicide target due to its essential role in histidine biosynthesis (Ames, 1957; Hilton et al., 1965). It catalyzes the Mn(II)-dependent dehydration of (2*R*,3*S*)-2,3-dihydroxy-3-(1H-imidazol-5-yl)propyl dihydrogen phosphate (2*R*3*S* IGP) to 3-(1H-imidazol-4-yl)-2-oxopropyl dihydrogen phosphate (IAP). A previous structure of IGPD isoform 1 from *Arabidopsis thaliana* (IGPD1) (PDB: 2F1D) (Glynn et al., 2005) and a second from *Mycobacterium tuberculosis* (PDB: 4GQU) (Ahangar et al., 2013) has shown that IGPD is a homo 24mer with two manganese ions, ∼6.5 Å apart, bound at each of the catalytic centers. These structures, and other biochemical data, have revealed that, unlike other enzymes, where Mn^2+^ can be exchanged with Mg^2+^ or Zn^2+^ with little effect on activity (Andreini et al., 2008), IGPD has both a structural and mechanistic requirement for manganese (Petersen et al., 1997). Analysis of the IGPD1 structure, coupled with the observation that triazole-phosphonate compounds are potent inhibitors of the enzyme (Hawkes et al., 1993; Jin et al., 2015; Lindell et al., 1996; Mori et al., 1995), has suggested that the reaction (Figure 1A) proceeds via an initial high-energy deprotonation of the substrate imidazole ring (p*K*_a_ ∼14.5) to yield an anionic imidazolate (Walba and Isensee, 1961), which is then converted to the product via diazafulvene and enol intermediates. Since the substrate lacks a carbonyl or imine adjacent to the leaving proton, which is thus non-acidic, IGPD uses an unusual mechanism for catalysis that is distinct from other dehydration reactions that have been reported to date (Glynn et al., 2005).

As part of a program of herbicide development designed to combat increasing resistance to glyphosate, we sought to expand our understanding of the enzyme mechanism by studying the structure of the IGPD in complex with its substrate, IGP, and also with inhibitors. In this paper, we describe two distinct crystal structures of the enzyme-substrate complex, one that represents initial capture of the substrate by the enzyme and a second in which the substrate is bound as the activated high-energy imidazolate form that is primed for catalysis. Analysis of the interconversion between the two forms reveals that progression through the catalytic cycle is a highly dynamic process involving conformational changes to both the substrate and the protein. Central to this process is the repeated switch in coordination state of an active site Mn(II) between six- and five-coordinate species. These different coordination states are linked to specific steps in the reaction and serve to control progression through the catalytic cycle. This study therefore explains how an enzyme can exploit a feature of transition metals to augment the functionality of proteins, providing insights into one of the roles that divalent metal ions can play in catalysis.

## Results

### The Form A Enzyme-Substrate Complex Contains a Ligand-Depleted Five-Coordinate Mn(II) Ion

**Figure 2:**
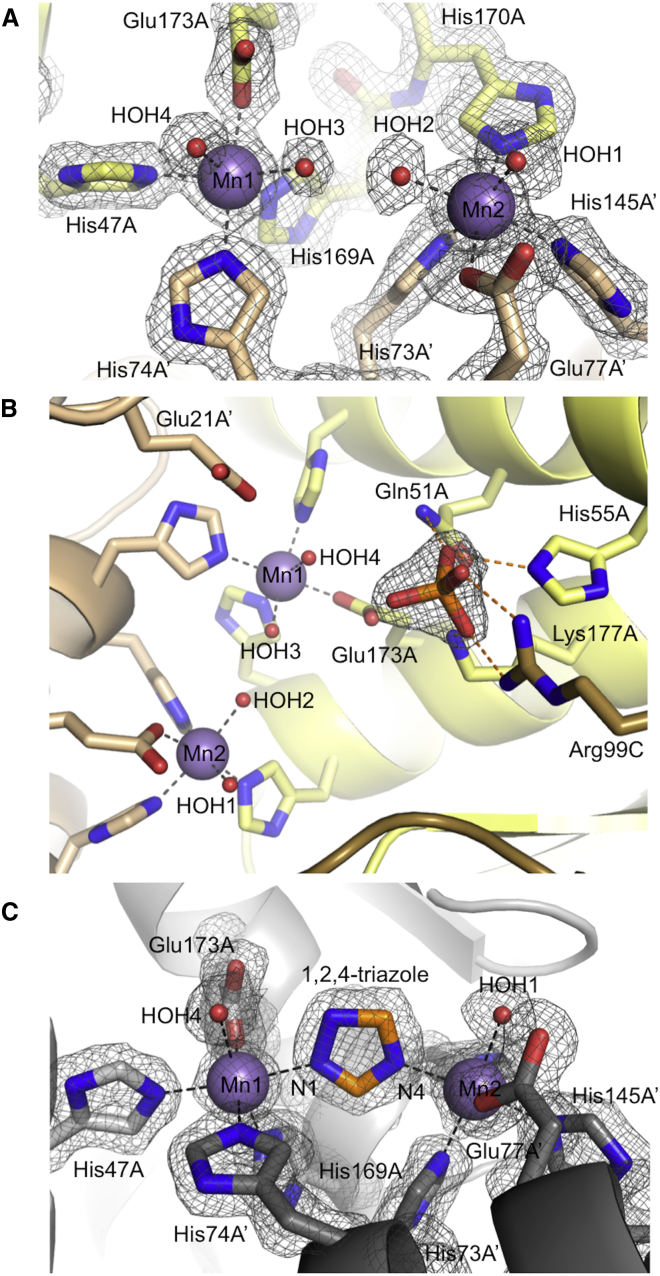
Mn Coordination and Ligand Binding in the Complexes of IGPD2 with Phosphate and 1,2,4-Triazole (A) In the *holo*enzyme structure, two manganese ions (Mn1 and Mn2) are octahedrally coordinated each by three histidine residues, a glutamate (from chains A and A′) and two water molecules (labeled HOH1-4). The 1.75-Å 2mFo-DFc electron density map (gray mesh) is shown surrounding the metal ions and their ligands, contoured at 1.2 σ. The protein backbone and side-chain carbon atoms are shown in yellow (chain A) or gold (chain A′), with other atoms in atom colors (N blue, O red, and P orange). The metal ions and selected water molecules are labeled and shown as purple or red spheres, respectively. Metal ion interactions are shown as black dashes. (B) Each active site of the 24mer is constructed at the interface between three chains, labeled A, A′, and C. The phosphate binding site is surrounded by a cluster of highly conserved basic residues (Gln51A, His55A, Lys177A, and Arg99C) that are hydrogen bonded (orange dashes) to the phosphate ion. The relative position of the manganese ions and the two putative catalytic residues (Glu21A and Glu173A) are also shown, with labels for the metal ligands omitted for clarity. The atom colors, metal interactions, and electron density are shown as in (A). See also Figure S2. (C) 1,2,4-Triazole (orange carbons) binds between the metal ions, each of which is octahedrally coordinated. The 1.3-Å 2mFo-DFc electron density map (gray mesh) surrounding the triazole is contoured at 1.5 σ. The protein backbone and side-chain carbon atoms are shown in silver with other atoms shown in atom colors as in (A). The manganese ions, coordinating waters, and metal ion interactions are labeled and shown as in (A).

To provide a foundation on which to analyze substrate binding, we determined the structure of *A. thaliana* IGPD isoform 2 (IGPD2) in complex with inorganic phosphate (1.75 Å resolution) (Table 1), which is a weak inhibitor of the enzyme (*K*_i_ ∼5 mM) (Wiater et al., 1971a). The native protein crystallized as the same 24mer species that has been observed previously (Figure S2), and this structure shows that within the active site of the *holo*enzyme, both of the Mn(II) ions are octahedrally coordinated by four conserved protein ligands (Figures 1B and S1) and two water molecules; HOH3/HOH4 for Mn1 and HOH1/HOH2, for Mn2 (Figure 2A). The structure also shows that a phosphate ion binds in a site surrounded by a number of conserved positively charged residues (Q51, H55, R99, and K177) (Figure 2B), which are equivalent to those that surround the site of a bound sulfate ion in the IGPD1 complex (PDB: 2F1D) (Glynn et al., 2005). The *holo*enzyme has an open conformation where the C-terminal region (R193–R206, termed the C loop), which contains a number of conserved residues, is disordered (Figures 1B and S1).

To study substrate binding in IGPD, we used site-directed mutagenesis to produce an inactive mutant of *A. thaliana* IGPD2 by replacing E21, a putative acid-base catalyst, with a glutamine residue. The E21Q IGPD2 mutant was confirmed to be inactive using a modified version of the stopped assay previously described by Ames (1957) and Hawkes et al. (1995), which showed no signal above background. E21Q IGPD2 was co-crystallized with substrate to yield a 1.12 Å resolution structure of the enzyme-substrate complex, termed form A (Table 1). The electron density clearly showed that the crystal contained a mixture of the enzyme bound to either the substrate, 2*R*3*S* IGP, or the 2*S*3*S* diastereoisomer, which is a by-product of the synthesis and is not a substrate of the enzyme (Hawkes et al., 1995; Saika et al., 1993) (Figure S3). Both diastereoisomers bind at the same site, with the enzyme adopting the same, open conformation equivalent to that of the *holo*enzyme, including the disordered C loop. The mode of binding of the substrate in the form A complex is comparable to that seen in the 2.1 Å resolution *Mycobacterium tuberculosis* IGPD-substrate complex (Ahangar et al., 2013), but the substantially increased resolution of the structure described here has enabled a much deeper analysis of how IGPD interacts with its substrate.

**Figure 3:**
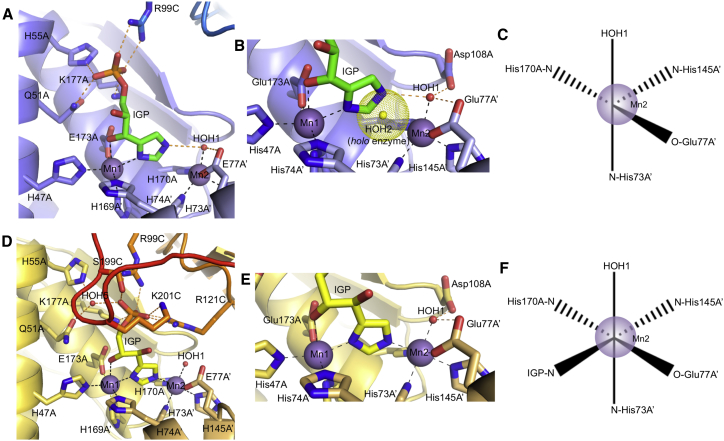
Comparison of the Mode of Binding of IGP in the Form A and Form B Complexes (A) An overview of the mode of binding of IGP in the form A complex. The protein backbone and side-chain carbon atoms are shown in a different shade of blue for each chain, and the substrate carbon atoms are shown in green. The non-carbon atoms, water molecules, metal ions, and metal and hydrogen bonding interactions are all drawn, labeled, and colored as in Figure 2. See also Figure S3. (B) Detail around the metal binding site in the form A complex, including the superimposed position of HOH2 from the *holo*enzyme complex (yellow sphere with dots representing the van der Waals radius). The binding of the neutral IGP-imidazole displaces HOH2 from the coordination sphere of Mn2, leaving the metal ion five-coordinate. Figure drawn as in (A). (C) A schematic drawing depicting the five ligands that coordinate Mn2 in the form A complex. The manganese ion is represented as a purple sphere. (D) An overview of the mode of binding of IGP in the form B complex. The protein backbone and side-chain carbon atoms are shown in a different shade of orange for each chain, the substrate carbon atoms are yellow, and the C loop is highlighted in red. The non-carbon atoms, water molecules, metal ions, and metal and hydrogen bonding interactions are all drawn, labeled, and colored as in Figure 2. See also Figure S4. (E) Detail around the metal binding site in the form B complex showing that the IGP-imidazolate is bound between the two Mn(II) ions, both of which are octahedrally coordinated. Figure drawn as in (D). (F) A schematic drawing depicting the octahedral metal coordination around Mn2 in the form B structure. The manganese ion is represented as a purple sphere.

In the form A complex, the 2*R*3*S* substrate forms a bidentate interaction with Mn1 via the imidazole-N1 nitrogen atom and the C3-OH group, which replace HOH3 and HOH4 as ligands to the metal ion, respectively (Figures 3A and 3B). Mn1 therefore retains the octahedral coordination seen in the *holo*enzyme complex. In contrast to Mn1, the second manganese ion, Mn2, is five-coordinate, sharing the same four protein ligands (E77, H145, H73, and H170) and one water ligand (HOH1) with those seen in the *holo*enzyme structure. However, the position of the imidazole-N3 nitrogen atom of the substrate is such that it would be too close to that of HOH2, the sixth Mn2 ligand in the *holo*enzyme. This potential steric clash in the form A complex is avoided by the prior displacement of HOH2 from the enzyme (Figure 3B). The substrate imidazole-N3 nitrogen atom forms a hydrogen bond to HOH1, which in turn is hydrogen bonded to two conserved carboxyl groups (E77 and D108). This arrangement implies that the imidazole ring is neutral, with its N3 nitrogen atom protonated and acting as a hydrogen bond donor, consistent with its high p*K*_a_ (∼14.5) for deprotonation. Compared with the *holo*enzyme structure, the position of Mn2 is shifted ∼0.5 Å out of the plane of the four metal ligands HOH1, O-E77, N-H73, and N-H170 (Figures 3B and 3C). This movement is also accompanied by a shortening of the mean metal-ligand distances by approximately 0.1 Å compared with other octahedrally coordinated Mn(II) ions in complexes of IGPD (Table 2). The structure of form A therefore shows that IGPD links the binding of its substrate to the generation of a ligand-depleted, five-coordinate Mn(II) ion at the heart of the active site.

### The Form B Enzyme-Substrate Complex Traps the Imidazolate Form of IGP

**Figure 4:**
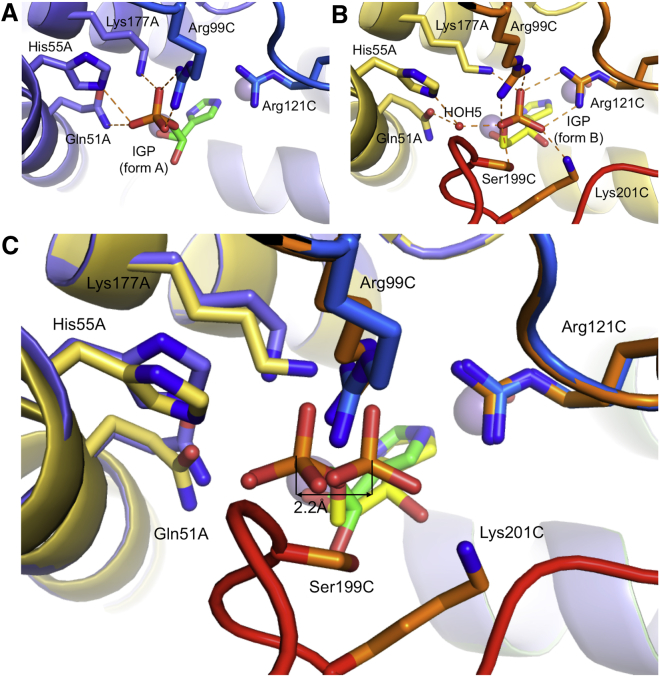
A Comparison of the Two Phosphate Binding Sites Observed in the Form A and Form B Complexes (A) The interactions between the phosphate group of the IGP (green carbons) and the enzyme in form A. Hydrogen bonds are shown as orange dashes. Representation of the metal ion, backbone, and side-chains colors is the same as in Figures 3A and 3B. (B) The interactions between the phosphate group of the IGP (yellow carbons) and the enzyme in form B. Hydrogen bonds are drawn as orange dashes. HOH5 is labeled and shown as a red sphere. Representation of the metal ion, backbone, and side-chains colors, including the red C loop, is the same as in Figures 3D and 3E. (C) A superposition of the structures shows that the ordering of the C loop (red) in form B repositions the phosphate group into a site that is ∼2.2 Å away from that in form A (double-headed arrow). There are also minor differences in the conformation of the side chains surrounding the two sites.

A second set of crystallization conditions yielded crystals in the same space group and with equivalent cell dimensions to those observed in form A. However, in the structure determined from these crystals (1.41 Å resolution) (Table 1), termed form B, both the enzyme and the substrate have strikingly different conformations to those seen in form A. In this structure, the C loop is ordered and buries the substrate from solvent, forming a closed conformation of the enzyme, which has not been reported previously (Figure 3D). The position of the C loop in form B occludes the site where an ethylene glycol molecule, originating from the crystallization precipitant, is located in form A. Unlike the situation in form A, where the active site is occupied by a mixture of two diastereoisomers of IGP, in form B the electron density could be explained by the binding of a single molecule of the 2*R*3*S* IGP substrate, with only a small number of disconnected, very low-level difference features seen around the substrate binding site (Figure S4). The conformational changes observed in the C loop cause the side chain of S199 to occlude the phosphate binding site observed in form A, resulting in the phosphate group of the IGP being repositioned in a new site, approximately 2.2 Å away from that seen in form A (Figure 4). This forces the substrate to adopt a radically different mode of binding, such that the IGP-phosphate group in form B makes new interactions with R121, S199, and K201, while maintaining those with R99 and K177, albeit with minor conformational changes to these side chains. Moreover, the shift in phosphate position is accompanied by major changes in the torsion angles of the substrate. While these changes do not affect the position of the imidazole-N1 atom within the coordination sphere of Mn1, they do result in the exchange of the C3-OH for the C2-OH as the second ligand to Mn1 (Figures 3D and 3E). This generates a different geometric arrangement around the metal ion and is accompanied by a shift in the position of the IGP-imidazole, which in form B sits equidistant between the two metal ions. Thus, the imidazole-N1 and -N3 nitrogen atoms interact directly with Mn1 and Mn2, respectively, with both of the metal ions octahedrally coordinated and lying in the plane of the imidazole ring (Figure 3F). This implies that both nitrogen atoms in the imidazole ring are deprotonated and that the ring is bound as an imidazolate anion, providing the first direct evidence for the existence of the imidazolate as an intermediate in the reaction. Furthermore, the conformation of the substrate is such that the C2, C3, and the imidazolate ring of the IGP lie in approximately the same plane, closely resembling the sp^2^ geometry of the carbon backbone in the proposed diazafulvene intermediate. The structure of form B therefore shows that when IGPD is in complex with the imidazolate intermediate, its active site is in a closed conformation and is solvent inaccessible, with both metal ions octahedrally coordinated.

As the C loop forms a crucial part of the active site in the form B complex, and since the conserved residues in this region form part of the phosphate binding site (Figure 4), we investigated whether the C loop was required for activity. A C-terminal deletion mutant (residues 170–176) was made in the IGPD homolog from *Pyrococcus furiosus* (*Pf*). Activity tests on the wild-type protein, using a modified version of a stopped assay protocol at 65°C (Ames, 1957; Hawkes et al., 1995) gave a specific activity of ∼50 μmol IAP mg^−1^ min^−1^. In contrast, no activity above background could be recorded for the ΔC-loop IGPD, demonstrating that the C loop is essential for catalysis.

### 1,2,4-Triazole Mimics the Binding of the Imidazolate

IGPD is inhibited by 1,2,4-triazole and 3-amino-1,2,4-triazole (amitrole) (Hilton et al., 1965; Wiater et al., 1971b), both of which have a chemical structure related to that of an imidazolate anion. To determine whether the mode of binding of 1,2,4-triazole resembles that of the substrate imidazolate seen in the form B structure, we co-crystallized wild-type IGPD2 with 1,2,4-triazole and obtained a 1.3 Å resolution structure of the complex (Table 1). The conformation of the enzyme in this structure was similar to that of the *holo*enzyme, including the disordered C loop, and a number of water molecules were seen to occupy the available hydrogen bonding positions within the phosphate binding site. Electron density between the two metal ions clearly showed that 1,2,4-triazole binds between the metal ions, with the N1 and N4 nitrogen atoms coordinating Mn1 and Mn2, respectively, replacing HOH3 and HOH2 from the *holo*enzyme structure (Figure 2C). Both of the manganese ions are therefore octahedrally coordinated and lie in the plane of the triazole ring. 1,2,4-Triazole binds in an equivalent way to that of the imidazolate in the form B complex, suggesting that inhibition of IGPD by 1,2,4-triazole is based on it mimicking the mode of binding of the high-energy imidazolate intermediate.

## Discussion

### Interconversion between the A and B Forms Reveals Novel Features of the IGPD Reaction Mechanism

**Figure 5:**
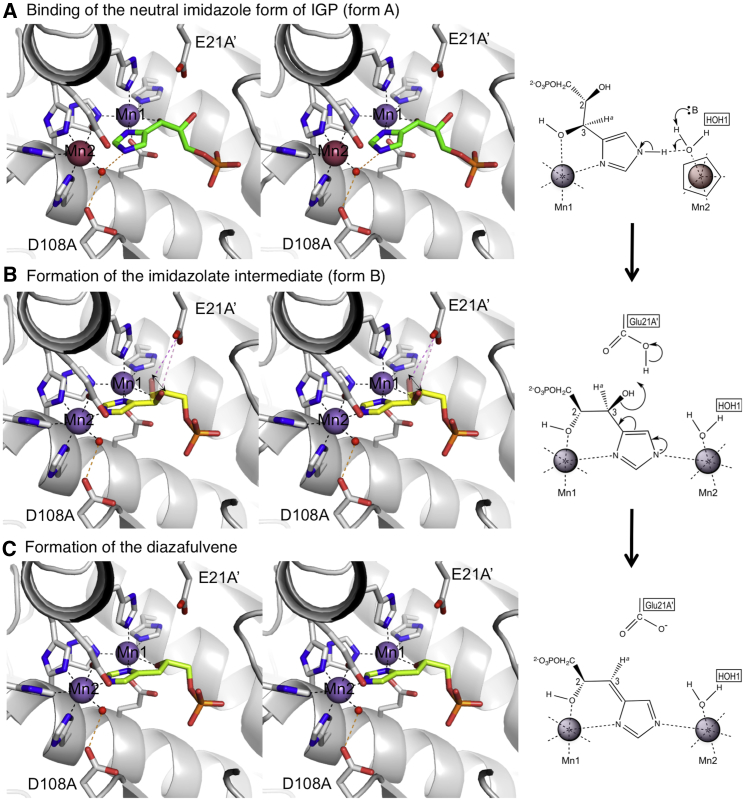
The Reaction Mechanism of IGPD (A) Stereo view to show the initial binding mode of the substrate to IGPD. IGP (green carbons) binds with a neutral imidazole ring, displacing HOH2 and leading to five-coordinate geometry around Mn2 (maroon sphere). The protonated imidazole-N3 atom forms a hydrogen bond (orange dashes) to HOH1 (red sphere), which is also hydrogen bonded to D108 and E77. The protein backbone and side-chain carbon atoms are shown in white; all non-carbon atoms are colored as in Figure 4. E21 is modeled on the basis of its position in the wild-type complexes, and other relevant side chains are shown as sticks, with labels omitted for clarity. Schematics representing the first two steps in the proposed reaction mechanism accompany the stereo views with the five-coordinate manganese ion, Mn2, colored maroon and enclosed in a pentagon. Schematic produced with ChemDraw. See also Movie S1. (B) Ordering of the C loop (not shown) induces a conformational change, triggering deprotonation of the IGP-imidazole to imidazolate (yellow carbons) and restoring the octahedral coordination of Mn2. This is accompanied by the exchange of the C3-OH for the C2-OH as the ligand to Mn1 and repositioning of the IGP-phosphate. During substrate rearrangement between the form A and form B states, the C3-OH is transiently perpendicular (partially transparent) to the imidazolate, which is required for C3-OH elimination. A double-headed arrow represents oscillation around the intermediate. E21 is within close proximity (3.3 Å) to the C3-OH (pink dashes). Figure is drawn as in (A). (C) The modeled position of the diazafulvene intermediate (lime green) in which the C3-OH has been eliminated. Figure is drawn as in (A).

We take the view that the two enzyme-substrate complexes presented here represent two different snapshots of the early steps in the reaction pathway of IGPD. The form A complex demonstrates how the open form of the enzyme can capture the neutral imidazole form of IGP. This is consistent with the expectation that, at biological pH, the neutral form of IGP would predominate over the imidazolate in solution by ∼10^7^-fold, given the p*K*_a_ of ∼14.5. In contrast, in form B the enzyme is in a closed conformation due to the ordered C loop. This is associated with the production of an imidazolate anion, the charge on which is critical for the subsequent dehydration step of the reaction, where pi-electron donation leads to loss of the C3 hydroxyl, facilitated by the weak electron-withdrawing nature of Mn(II). The form B complex thus represents a trapped intermediate state of the reaction. The interconversion of the form A and form B structures therefore describes progress through the reaction, in a process that is clearly associated with a number of dramatic torsion angle changes to the IGP, the switch in coordination chemistry at Mn2, and the ordering of the C loop. We presume that these structural changes reflect the molecular processes that dominate the early steps in catalysis, and to investigate the processes that facilitate production of the imidazolate we have analyzed the conformational changes that occur during interconversion between the open and closed states of the enzyme-substrate complex (Figure 5 and Movie S1). In the following sections, we analyze the implications of this for the mechanism of the enzyme.

### Electrostatic and Kinetic Roles for the Manganese Ions in Catalysis

The structures described here clearly point to two critical electrostatic roles for the manganese ions in facilitating the deprotonation of the imidazole during the first step in catalysis. In the form A structure, where the imidazole-N1 nitrogen atom ligates Mn1 and the N3 nitrogen is hydrogen bonded to HOH1 (Figure 3B), the electron-withdrawing effect of Mn1 is likely to reduce the p*K*_a_ of the protonated imidazole-N3 nitrogen atom. This suggests that the way that the substrate interacts with Mn1 during the initial binding phase of the reaction lowers the energy barrier to deprotonation of N3 and formation of the imidazolate. Analysis of the form B structure provides evidence for a second electrostatic role for the manganese ions. In this structure, the mode of binding of the negatively charged imidazolate between the pair of manganese ions results in the metal ions becoming shielded from the repulsion that they exert on each other (Figure 3E). The pair of manganese ions in IGPD therefore acts as a coulombic trap for the negatively charged imidazolate intermediate.

The form B structure also demands that as the substrate imidazole is converted to an imidazolate, its N3 nitrogen occupies the position of HOH2 within the coordination sphere of Mn2 in the *holo*enzyme. However, restrictions arising from the shape of the active site require that the direction of approach to Mn2 of the incoming imidazolate-N3 atom would be the same as that of an outgoing water molecule, creating a steric problem that the enzyme must resolve. Analysis of the form A structure provides an elegant explanation as to how the enzyme provides a sterically unopposed route for the capture of the imidazolate. When the enzyme binds the substrate, HOH2 is ejected from the active site and Mn2 is rendered five-coordinate. This leaves a vacant ligand position at Mn2 for the imidazolate to occupy once it has been formed, which simultaneously restores the octahedral coordination of this metal ion. Moreover, the form A complex shows that the imidazole-N3 atom forms a hydrogen bond to HOH1 (Figure 5A), which places this water molecule in an ideal position to act as a base for removal of the proton from N3. This arrangement shows that, together with the conserved carboxyls (E77 and D108) that are hydrogen bonded to HOH1, the active site contains a proton relay system that provides a path to solvent for the departing proton. Taken together, these data suggest that the binding mode of the substrate primes the IGPD active site for catalysis. This is critically dependent on the generation of the five-coordinate manganese ion, which solves the kinetic problem of providing a catalytically productive pathway.

### The Five-Coordinate Mn(II) Ion Leads to Weak Substrate Binding in IGPD

In the reaction, the equilibrium between the imidazole/five-coordinate Mn2 ion and the imidazolate/six-coordinate Mn2 ion is presumably significantly shifted compared with the deprotonation of imidazole to imidazolate in water. Although it is not possible to reliably estimate the free energy changes associated with generating a five-coordinate manganese ion, pentacoordinate-Mn complexes have only been observed in metalloenzymes associated with changes in redox state of Mn(II) to Mn(III), such as oxalate oxidase and superoxide dismutase (Barynin et al., 2001; Edwards et al., 1998). In the IGPD reaction there is no change in the redox state of the manganese and, thus, the switch in coordination from six to five seems likely to incur an energy penalty. In *A. thaliana* IGPD2 the *K*_m_ for IGP (170 μM) is rather high (Figure S5), despite numerous stabilizing interactions formed between the substrate and the enzyme in the form A complex (Figure 3A). This suggests that, unless the following catalytic step is very fast, the weak binding of the substrate could be explained by the energy penalty incurred by the generation of the five-coordinate metal center at Mn2.

### The C Loop Triggers Conformational Changes that Are Critical for Diazafulvene Production

Comparison of the form A and form B structures shows that there is a disorder/order transition of the C loop during the reaction. Since the position of the ordered C loop in form B is incompatible with the position of the phosphate group of IGP in form A, we propose that ordering of the C loop triggers the movement of the phosphate group of the IGP from the form A site to the form B site during the reaction. This shift in the phosphate is accompanied by changes to the IGP torsion angles and an exchange of the C3-OH of IGP for the C2-OH as a ligand to Mn1. We have modeled these torsion angle changes to determine the optimal path of rotation (Movie S1). The only movement that avoids potential steric hindrance between the C3-OH and other residues in the active site involves the C2-OH and C3-OH passing each other as they move toward, and away from, Mn1, respectively. During this rotation the C3-OH is transiently perpendicular to the imidazolate ring, maximizing the overlap of its anti-bonding orbital with the pi-electron system of the ring. This is the exact requirement for the geometry of the optimal transition state that leads to the loss of the C3 hydroxyl from the imidazolate intermediate, to form the diazafulvene intermediate (Nasipuri, 1991). When the C3-OH is perpendicular to the imidazolate ring it is within 3.3 Å of one of the carboxylate oxygens of E21 (Figure 5B), which is the proposed acid-base catalyst involved in diazafulvene formation. Furthermore, oscillation of the substrate torsion angles around a form B-like structure would allow this transition state to be accessed at a higher frequency, favoring production of the diazafulvene (Figure 5C).

### The Form B Structure Indicates How IGPD Catalyzes the Conversion of the Diazafulvene to IAP

In the next stage of the reaction, the diazafulvene is converted to an enolic intermediate by abstraction of the C2 proton, prior to an enzyme-catalyzed tautomerization that leads to the product, IAP (Glynn et al., 2005). The similarity of the mode of binding of IGP in the form B structure to a diazafulvene allows us to examine conversion to the enol intermediate and the final product, IAP. In form B, the C2-OH of the IGP ligates Mn1 and, thus, the C2-H is rendered more acidic due to its proximity to the metal ion. This would favor the loss of the proton from the diazafulvene during the reaction, in a process that is likely to be catalyzed by E173, the carboxyl of which is immediately adjacent to the C2 (3.5 Å) (Movie S1). This residue is also conveniently placed to participate in the enzyme-catalyzed tautomerization of the enol to IAP.

### Switching Coordination Chemistry: Understanding the Role of Specific Metal Ions in Controlling Enzyme Catalysis

The studies described here provide a molecular explanation of the unusual dehydration mechanism catalyzed by IGPD. Central to this is the enzyme-catalyzed formation of a high-energy imidazolate intermediate, without which the reaction rate at pH 7 would be limited by the rate of productive substrate binding. We have demonstrated how changes in manganese coordination chemistry dominate all aspects of catalysis in IGPD. In the first part of this process, the enzyme harnesses the substrate binding energy to create a distorted, ligand-depleted metal center, which serves to remove kinetic barriers to the production of the imidazolate intermediate. Subsequently, a second switch in coordination chemistry restores the octahedral coordination of the metal ion, leading to critical torsion angle changes to the substrate that are essential for concomitant production of the diazafulvene. More widely in biology, the loss of a ligand from an octahedrally coordinated metal ion is not without precedence, for example the changes in coordination state of iron in both soluble methane monooxygenase (Rosenzweig et al., 1993) and hemoglobin (Perutz, 1970). Although five-coordinate Mn(II) centers and changes in coordination states have also been observed in the structures of both manganese superoxide dismutase and oxalate decarboxylase (Tabares et al., 2005, 2009) their roles, unlike IGPD, are associated with changes in metal redox state during the reaction. To our knowledge, the work on IGPD presented here is the first description of how an enzyme can exploit the unique ability of transition metals to switch coordination states at specific steps in catalysis. In IGPD this facilitates a highly choreographed and dynamic process that controls the reaction mechanism at every stage. This role of metal ions may well be a more important driver to catalysis than is currently recognized.

## Experimental Procedures

### Cloning and Mutagenesis

Two ΔN constructs of the gene encoding for IGPD2 from *A. thaliana* were designed to remove an N-terminal signal peptide and cloned into pET24a vectors (Novagen). Construct A (residues 54–272), was provided as a synthetic gene (GeneArt) and construct B (residues 69–272), which was further truncated at the N terminus, was cloned from the original construct. The E21Q mutant was made by introducing a point mutation to construct B by QuickChange site-directed mutagenesis (Stratagene), subsequently verified by sequencing (GeneServices). The IGPD2 complex with phosphate was determined from crystals grown with construct A, while the 1,2,4-triazole complex was determined using construct B. The gene encoding for IGPD from *Pf* was PCR amplified with a mutation to the start codon, which replaced a GTG with an ATG, before ligation into pETBlue (Novagen). A ΔC construct of the *Pf* enzyme was also cloned from the full-length gene, introducing a premature stop codon, which removed the C-terminal loop region of the protein (residues 170–176). The ΔC construct was subsequently cloned into a pET24a vector for protein expression.

### Expression and Purification

Plasmids encoding for IGPD2 were transformed into *Escherichia coli* BL21(DE3) cells (Novagen) and protein expression was induced for 5 hr with 1 mM isopropyl-β-D-thiogalactopyranoside at 37°C in Luria broth, supplemented with 5 mM MnCl_2_. Plasmids encoding for the *Pf* IGPD were transformed into Tuner DE3 cells (Novagen) and expressed using the same method, but for 3 hr and supplemented with 4 mM MnCl_2_. Cells were harvested and lysed by sonication. For IGPD2, insoluble material was removed by centrifugation and the cell-free extract was purified in a process involving anion-exchange, hydrophobic, and size-exclusion chromatography, combined with ammonium sulfate precipitation. For *Pf* IGPD, cell-free extract was heated to 70°C for 20 min, before purification using anion-exchange and size-exclusion chromatography. All proteins were analyzed after each stage by SDS-PAGE.

### Crystallization and Data Collection

Prior to crystallization, substrate (Toronto Research) or inhibitor (Sigma) were added to the protein (10 mg ml^−1^ in 50 mM Bis-Tris propane buffer [pH 8.0], 50 mM NaCl, 10 mM β-mercaptoethanol, and 0.4 mM MnCl_2_) at a final concentration of 5 mM. Initial crystallization conditions were determined by automated screening (Nextal) using a Matrix Hydra II crystallization robot. Crystals were optimized by hanging-drop vapor diffusion using a 5:1 ratio of protein to precipitant, which yielded cubic crystals after 5–10 days when equilibrated against a 1-ml reservoir of precipitant at a temperature of 17°C. Crystals were cryoprotected in their crystallization solution containing 25%–30% ethylene glycol, prior to flash-cooling in liquid nitrogen. All data sets were collected on the MX beamlines at the Diamond Light Source. The two mutant complexes with IGP and the 1,2,4-triazole complex belonged to the space group P432 with cell dimensions of *a* ≈ 113 Å. The structure with phosphate was determined to be in the space group I23 with cell dimensions of *a* = 225 Å. The data collection statistics are summarized in Table 1.

### Structure Determination and Refinement

Data were processed using Xia2 (Winter, 2010) or by integration using iMosflm (Leslie and Powell, 2007). All additional data processing was carried out with CCP4i (Winn et al., 2011). The IGPD2 complex with phosphate was determined by molecular replacement using PHASER (McCoy et al., 2007) with a monomer of *A. thaliana* IGPD1 (PDB: 2F1D) (Glynn et al., 2005) as a search model (sequence identity ∼96%). The 1,2,4-triazole complex was determined by the same method, using the refined protein coordinates from the phosphate complex as a search model. All other structures were directly refined using the protein atoms from the phosphate complex, but omitting ligands, metal ions, and solvent. Model building and refinement was carried out in Coot (Emsley and Cowtan, 2004) and Refmac5 (Murshudov et al., 1997), with ligand libraries generated by JLigand (Lebedev et al., 2012). Refinement statistics are summarized in Table 1. For all structures, no electron density was visible for the residues before S75, which was renumbered S9 for consistency with the structure of IGPD1 from *A. thaliana* (PDB: 2F1D) (Glynn et al., 2005). Models were validated using MolProbity (Chen et al., 2010) and figures were generated using Pymol (Shrodinger, LLC).

### Determining the *K*_m_ of IGP Binding to IGPD2

The *K*_m_ was determined using a modified coupled assay (Hawkes et al., 1995). Assay buffer was combined with bovine glutamate dehydrogenase (Sigma) and a large excess of imidazoleacetol-phosphate transaminase (S. Singh, Syngenta). Reduced nicotinamide adenine dinucleotide (Sigma) and IGPD2 were added to a final concentration of 6 mM and 45 nM, respectively, before incubation at 30°C in a temperature-controlled spectrophotometer. When the signal at 340 nm had stabilized, IGP was added at various concentrations to start the reaction. Initial rates from the linear part of the curve were recorded, and *K*_m_ and *K*_cat_ were determined by non-linear least-squares fitting in GraphPad Prism.

## Author Contributions

C.B. and K.L.B. conducted the work on *A. thaliana* IGPD2 and, together with D.W.R. and P.J.B., interpreted the data and wrote the manuscript. R.C.V. conducted modeling studies and, together with T.R.H., contributed to discussions on the enzyme mechanism. S.E.S. established the purification procedures for the enzymes; T.C.E. conducted studies on *P. furiosus* IGPD; and H.F.R. provided technical assistance.

## Figures and Tables

**Table 1:** Data Collection and Refinement Statistics

	IGPD2+Pi (PDB: 4QNK)	IGPD2+1,2,4-Triazole (PDB: 4MU0)	E21Q IGPD2 Form A + IGP (PDB: 4MU3)	E21Q IGPD2 Form B + IGP (PDB: 4MU4)
**Data Collection**				

Beamline	Diamond I02	Diamond I24	Diamond I02	Diamond I24
Wavelength (Å)	0.9507	0.9686	0.9794	0.9686
Space group and unit cell parameters (Å) *a* = *b* = *c* =	I23	P432	P432	P432
	225.13	113.1	113.1	112.9
Molecules per asymmetric unit	8	1	1	1
Resolution (Å)	71.19–1.75 (1.84–1.75)	50.59–1.3 (1.33–1.3)	46.17–1.12 (1.14–1.12)	65.18–1.41 (1.45–1.41)
Unique observations	187811 (27,248)	61,097 (4421)	94,992 (6558)	47,547 (3450)
*R*_merge_^a^	0.118 (0.579)	0.053 (0.762)	0.041 (0.568)	0.085 (0.849)
*R*_pim_^b^	0.062 (0.325)	0.023 (0.324)	0.016 (0.303)	0.029 (0.282)
Mean((*I*)/SD(*I*))	11.7 (2.2)	25.2 (3.5)	30.8 (2.5)	17.7 (3.4)
Completeness (%)	99.4 (99.4)	100.0 (99.9)	99.1 (93.9)	100.0 (100.0)
Multiplicity	4.2 (4.0)	12.3 (12.5)	9.5 (4.7)	10.5 (10.8)

**Refinement**				

No. of non-H atoms	13,061	1654	1753	1818
R factor/*R*_free_^c^ (%)	13.3 (22.0)/18.9 (30.1)	13.0 (23.2)/14.8 (25.2)	12.2 (23.1)/13.8 (23.0)	12.4 (18.60)/15.4 (23.1)
Average B factors (Å^2^)	20	15	15	16
Bond length rmsd (Å)	0.013	0.011	0.012	0.012
Bond angle rmsd (°)	1.5	1.5	1.8	1.5
Ramachandran values	1431/1481 favored	187/193 favored	187/194 favored	203/209 favored
	50 allowed	6 allowed	6 allowed	6 allowed

Values in parentheses are data in the highest-resolution shell. Rmsd, root-mean-square deviation.

a *R*_merge_ = Σ_*hkl*_ Σ_i_ | *I*_i_ – *I*_m_|/Σ_*hkl*_ Σ_i_*I*_i_.

b *R*_pim_ = Σ_*hkl*_ √1/*n* − 1Σ_*i*=1_ | *I*_*i*_ − *I*_m_|/Σ_*hkl*_ Σ_*i*_*I*_i,_ where *I*_i_ and *I*_m_ are the observed intensity and mean intensity of related reflections, respectively.

c R factor = (Σ||*F*_o_| − |*F*_c_||/Σ|*F*_o_|) × 100, where *F*_o_ and *F*_c_ are observed and calculated structure factor amplitudes. *R*_free_ is calculated using 5% of reflections omitted from refinement.

**Table 2:** Average Metal-Ligand Distances (Å)

	IGPD2 + Pi^e^	IGPD2+ 1,2,4-Triazole	E21Q IGPD2 Form A + IGP		E21Q IGPD2 Form B + IGP
*Mn1 coordination number*	*6*	*6*	*6*		*6*
HOH3 or equivalent^a^	2.30 (2.22–2.47)	2.26	2.35		2.30
HOH4 or equivalent^b^	2.42 (2.36–2.47)	2.29	2.41		2.33
OE1 Glu173	2.19 (2.15–2.23)	2.17	2.15		2.16
NE2 His47	2.27 (2.22–2.29)	2.29	2.25		2.26
NE2 His169	2.24 (2.20–2.30)	2.31	2.26		2.30
NE2 His74	2.25 (2.22–2.28)	2.30	2.26		2.30
Mean	2.27	2.27	2.28		2.28
*Mn2 coordination number*^c^	*6*	*6*	*6*	*5*	*6*
HOH1	2.20 (2.13–2.26)	2.27	2.06	2.19	2.21
HOH2 or equivalent^d^	2.25 (2.20–2.26)	2.25	2.29	–	2.18
NE2 His170	2.24 (2.22–2.27)	2.27	2.28	2.16	2.29
NE2 His145	2.28 (2.22–2.32)	2.31	2.45	1.99	2.31
NE2 His73	2.24 (2.20–2.26)	2.25	2.19	2.19	2.24
OE1 Glu77	2.32 (2.28–2.35)	2.30	2.33	2.33	2.40
Mean	2.25	2.28	2.26	2.17	2.27

a Equivalents are either the IGP-imidazole, IGP-imidazolate, or 1,2,4-triazole N1 atom.

b Equivalents are either the C2-OH or C3-OH substituents of IGP.

c The ligand distance around Mn2 for the two diastereoisomers of IGP in the form A structure are shown separately.

d Equivalent is the 1,2,4-triazole N4 atom.

e Mean distances across all eight monomers in the asymmetric unit; range of measured distances is shown in parentheses.

## Accession Numbers

The atomic coordinates and structure factors have been deposited in the PDB under accession codes PDB: 4MU0, 4MU3, 4MU4, and 4QNK.

## Supplemental Information

Document S1. Figures S1–S5 and Supplemental Experimental Procedures

Movie S1. The Reaction Mechanism of IGPD

Document S2. Article plus Supplemental Information
