# Supplementary material for: Crystal Structures Reveal that the Reaction Mechanism of Imidazoleglycerol-Phosphate Dehydratase Is Controlled by Switching Mn(II) Coordination
Source: Structure. 2015 Jul 7;23(7):1236–45. doi: 10.1016/j.str.2015.05.012 (PMC4509728; doi:10.1016/j.str.2015.05.012)
Supplement: Document S1. Figures S1–S5 and Supplemental Experimental Procedures [file mmc1.pdf]

Structure, Volume *23*

Supplemental Information

Crystal Structures Reveal that the Reaction Mechanism of Imidazoleglycerol-Phosphate Dehydratase Is Controlled by Switching Mn(II) Coordination

Claudine Bisson, K. Linda Britton, Svetlana E. Sedelnikova, H. Fiona Rodgers, Thomas C. Eadsforth, Russell C. Viner, Tim R. Hawkes, Patrick J. Baker, and David W. Rice

Supplemental Data:

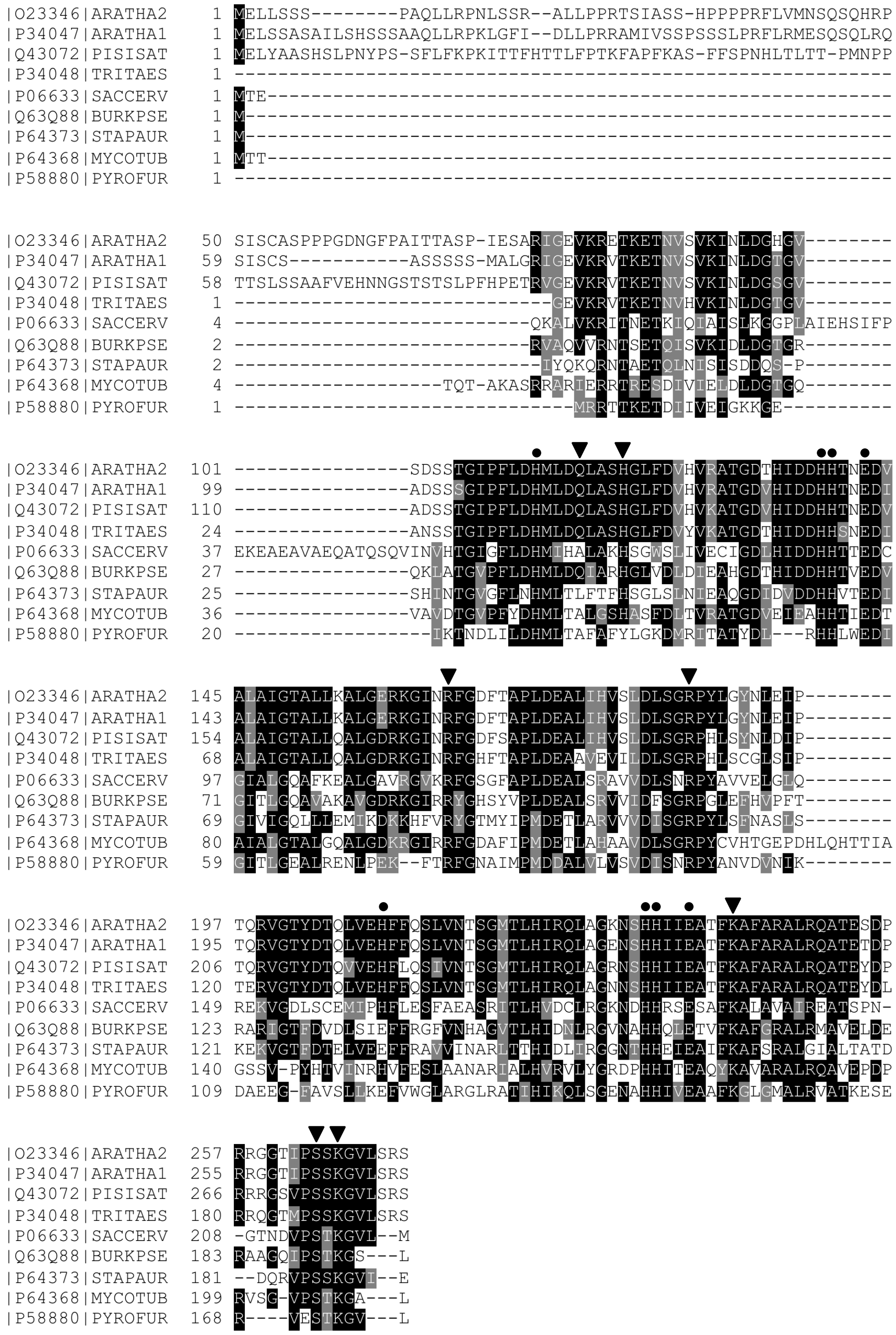

Figure S1. Related to Figure 1. Multiple sequence alignment of IGPD homologues from a range of species.

Examples are from *Arabidopsis thaliana* (ARATHA2; isoform 2 and ARATHA1; isoform 1), *Pisium sativum* (PISISAT), *Triticum aestivum* (TRITAES), *Saccharomyces cerevisae* (SACCERV), *Burkholderia pseudomallei* (BURKPSE), *Staphycoccus aureus* (STAPAUR), *Mycobacterium tuberculosis* (MYCOTUB) and *Pyrococcus furiosus*

(PYROFUR). Sequences are numbered according to the full-length polypeptide, including the N-terminal signal peptides for the plant homologues. Black and gray shading indicates identical or similar residues, respectively. Black dots indicate residues that are involved in metal ion binding. Residues implicated in recognition of the substrate-phosphate are marked with a black triangle. Sequences were retrieved from UniProt, the alignment was produced using Tcoffee and the figure was drawn using boxshade.

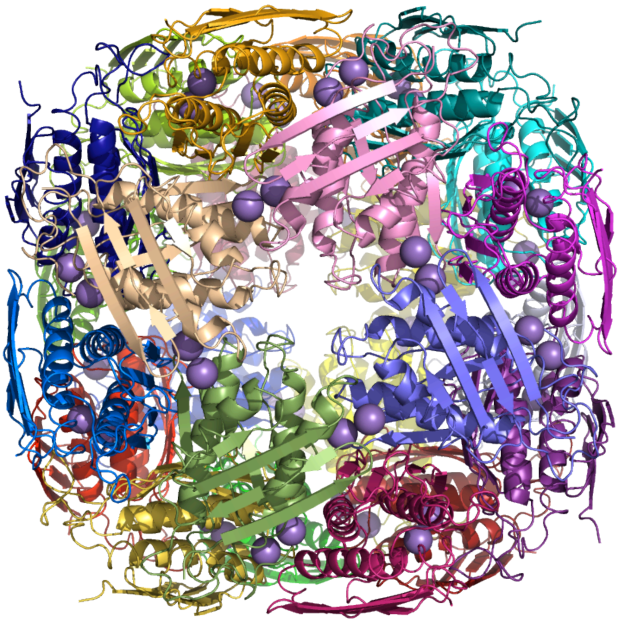

Figure S2. Related to Figure 2. The biological assembly of IGPD2. The 24mer is viewed down the 4-fold axis, chains are represented as cartoons and each is drawn in a different colour. The pair of Mn^{2+} ions in each active site are coloured purple and represented as spheres.

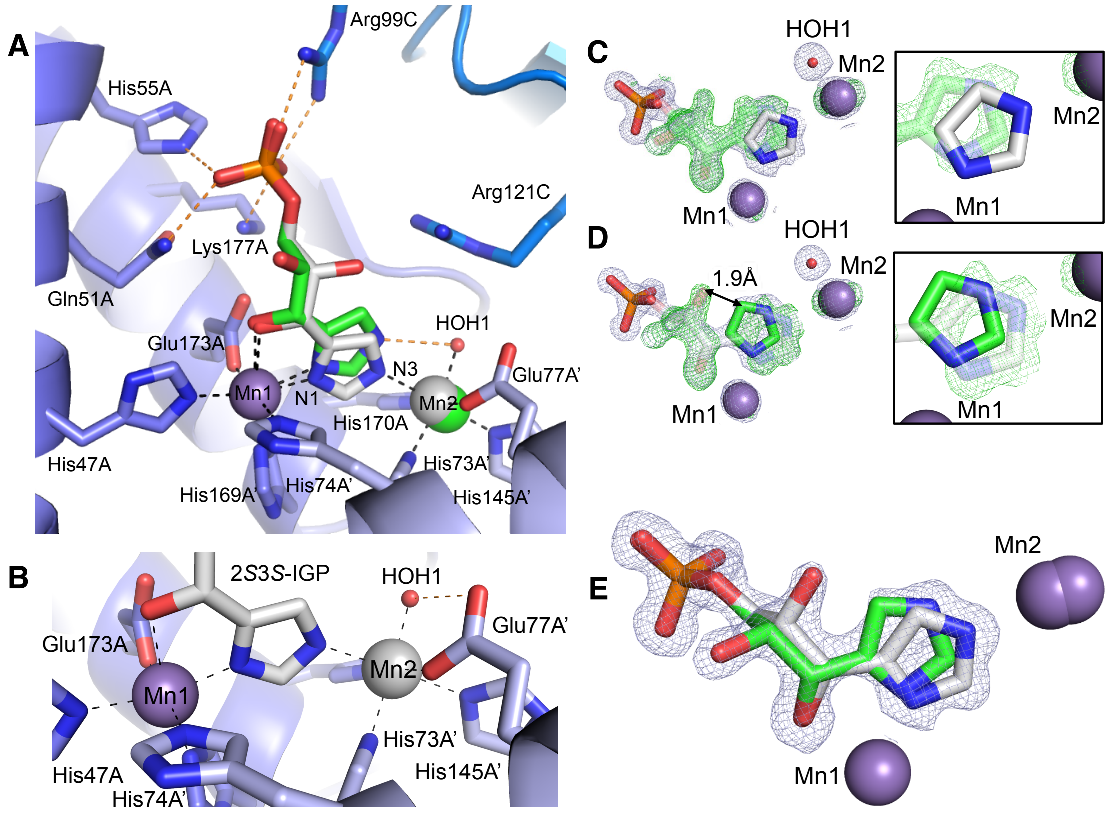

Figure S3. Related to Figure 3A-C. The mode of binding of the 2*R*3*S* and 2*S*3*S* diastereoisomers of IGP in the form A complex.

(**A**) Both the substrate, 2*R*3*S* IGP, (green) and the contaminant, 2*S*3*S* IGP, (gray) form a bidentate interaction with Mn1 via their imidazol(at)e N1 nitrogen and C3-OH atoms. The phosphate groups of both diastereoisomers bind in an equivalent position to each other and to that of the phosphate moiety observed in the *holo* enzyme complex. 2*S*3*S* IGP binds between the metal ions with an imidazolate anion whereas the substrate, 2*R*3*S* IGP, binds with a neutral imidazole. This leads to two distinct positions for Mn2, one octahedral (gray), associated with the 2*S*3*S* IGP and one square pyramidal (green), associated with the substrate. Important hydrogen bonds and metal ion interactions are shown as orange and black dashes, respectively.

(**B**) Diagram to show the interactions made between the metal ions and the 2*S*3*S* IGP. The imidazole ring is deprotonated, forming an imidazolate anion that interacts with both metal ions, each of which is octahedral.

(**C**) An imidazolate anion (gray) (0.4 occupancy) and a phosphate anion (occupancy

1.0) were modelled into the electron density map and refined. The resulting difference map (green mesh) revealed a second ring position (inset), which was interpreted as an imidazole based on the pattern of hydrogen bonding. The final position of the substrate (green) is shown partially transparent. The difference map is contoured at 4.0 σ and the 2mFo-DFc map (gray mesh) is contoured at 1.0 σ.

(**D**) Refinement of the imidazole (green) (0.6 occupancy) and a phosphate anion (1.0 occupancy) revealed difference density for the imidazolate anion that had been modelled previously (inset). Residual density in the difference maps (green mesh) indicated two positions corresponding to two hydroxyl groups due to the different chirality at C2. A steric clash between the 2*S* position of the hydroxyl and the C4 atom of the neutral imidazole (1.9Å) (black arrow) allowed for the 2*R* hydroxyl to be matched to the imidazole and the 2*S* hydroxyl to the imidazolate. The observation of positive difference density around Mn2 in both difference maps indicated that there were two positions for the metal ion. The final position of 2*S*3*S* IGP (gray) is shown partially transparent. The difference map is contoured at 4.0 σ and the 2mFo-DFc map (gray mesh) is contoured at 0.9 σ.

(**E**) The 1.15 Å 2mFo-DFc map (gray mesh) after refinement with 2*R*3*S* IGP and 2*S*3*S* IGP with the ligands modelled at occupancies of 0.6 and 0.4, respectively, is shown at 1.5 σ.

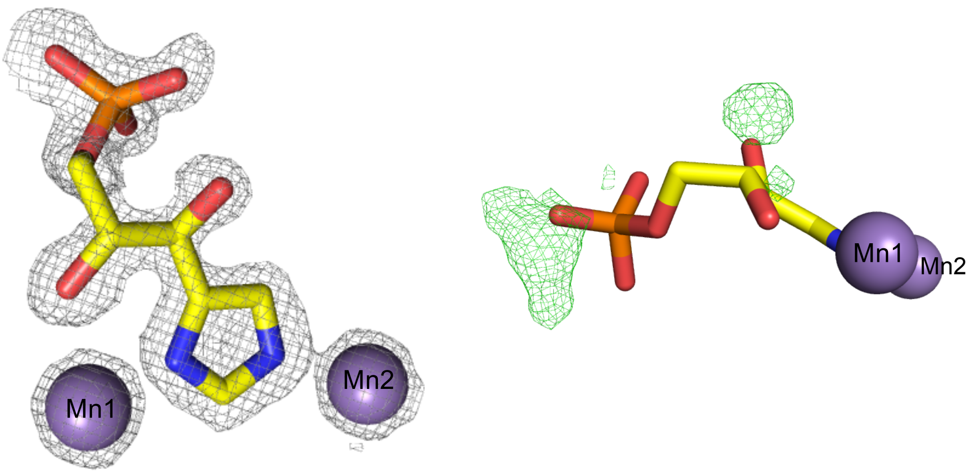

Figure S4. Related to Figure 3D-E. The mode of binding of 2*R*3*S* IGP in the form B complex.

The 1.4 Å 2mFo-DFc electron density map (gray mesh) is shown surrounding the modelled substrate at 1.5 σ. After refinement some low-level density features could be observed in the difference map, suggesting the binding of other minor species, but it was not possible to interpret the density. The difference map is shown in green at 4.5 σ. The IGP is coloured as in Figure 3D-E. The two manganese ions are labelled and shown as purple spheres.

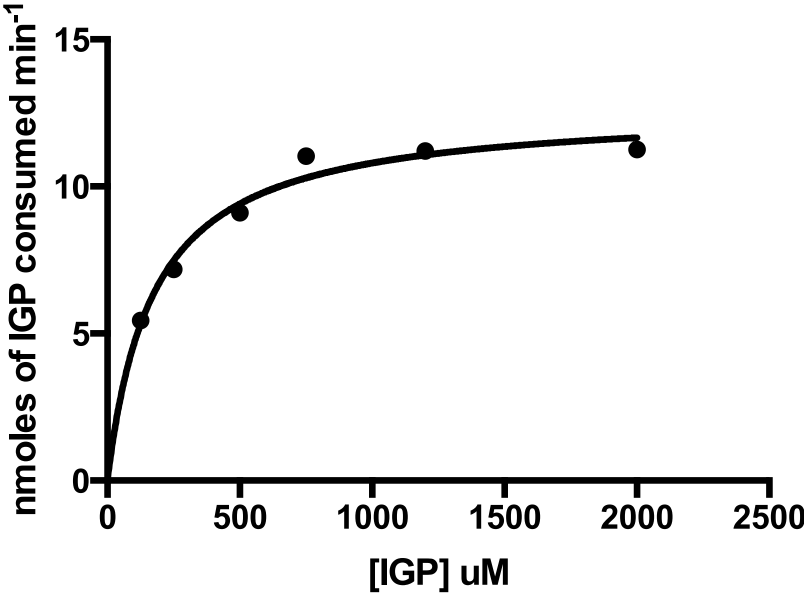

Figure S5. Related to experimental procedure: *Determining the Km of IGP binding to IGPD2.*

The Km was determined over various IGP concentrations from 125 μM to 2 mM using a coupled assay (Hawkes et al., 1995). After a period of lag, initial rates were recorded and plotted against the substrate concentration. The Vmax was calculated to be 12 nmoles (+/- 2 nmoles) of IGP consumed min-1 and the Km was estimated to be 170 μM (+/- 70 μM). Km and Kcat were determined by non-linear least squares fitting in Graph Pad Prism.

Movie S1. Related to Figure 5. The reaction mechanism of IGPD. A model of the reaction mechanism was produced using Pymol (Schrodinger, LLC) and interpolation between the form A and form B complex structure using LSQMAN (Kleywegt et al., 2001) from the Uppsala crystallography software factory (USF).

Supplemental Experimental Procedures:

Primer Name	***Sequence 5'>3'***
IGPD2-ΔN_f	CATATGGAATCAGCTCGAATAGGTGAAGTC
IGPD2-ΔN_r	GGATCCTTATGAACGTGACAAGACTCCTTTC
E21Q_sense	GAAGAGAGAAACAAAGCAAACAAATGTATCAGTGAAG
E21Q_antisense	CTTCACTGATACATTTGTTTGCTTTGTTTCTCTCTTC
Pf-IGPD_f	ATGAGGAGAACTACAAAAGAAACG
Pf-IGPD_r	TTACAATACCCCCTTTGTGCT
Pf-IGPD-ΔC_f	CATATGAGGAGAACTACAAAAGAAACG
Pf-IGPD-ΔC_r	GGATCCTTAAACTCTCTCACTCTCTTTAG

Construct Design

Two N-terminally truncated constructs of the *HISN5B* gene from *Arabidopsis thaliana* were designed in order to remove the N-terminal signal peptide from the over-expression product. The longer length construct, construct A (residues 54-272), was provided as a synthetic gene (GeneArt) and subsequently ligated into a pET24a (Novagen) expression plasmid using NdeI and BamHI restriction sites. The shorter construct, construct B (residues 69-272), which had a further 16 residues deleted from the N-terminus to completely remove the signal peptide, was cloned from the original construct using the pair of synthetic oligonucleotides, IGPD2-ΔN_f and IGPD2-ΔN_r, before ligation into a pET24a plasmid using NdeI and BamHI restriction sites. The IGPD2 complex with phosphate was determined from crystals grown with construct A, whilst the mutant complexes with substrate and the 1,2,4-triazole complex were determined using construct B.

Mutagenesis

Template plasmid encoding for construct B was propagated in *Escherichia coli* sub-cloning strain DH5α (Invitrogen) and purified by mini-prep (Qiagen). A pair of synthetic oligonucleotides, E21Q_sense and E21Q_antisense (Eurofins MWG Operon), were used to create an E21Q mutation using the Stratagene QuikChange site directed

mutagenesis procedure. The reaction was carried out in a 25 µl volume using an 18 cycle thermal cycling reaction that incorporated a 12 min elongation step. The template plasmid was then degraded by incubation with DpnI for 1 hr at 30 $^{\circ}$C and the resulting plasmid was transformed into XL1-Blue *E. coli* cells (Stratagene). Plasmid was harvested and sequenced (UoS Geneservice) to validate the mutation.

Protein over-expression and purification

Both the wild-type and mutant IGPD2 proteins were over-expressed in *E. coli* strain BL21 (DE3) (Novagen). After incubation at 37 °C for 5 hr post-addition of 1 mM IPTG and 5mM $MnCl_2$, cells were harvested by centrifugation, re-suspended in buffer A (50 mM Tris-HCl pH 8 and 50 mM NaCl) and lysed by sonication (2 x 20 s cycles at 16 micron amplitude on a Soniprep 150 sonicator). The lysate was centrifuged at 70000 g for 10 min to pellet the insoluble material and the cell free extract was applied to a 5 ml Hi-Trap DEAE-Sepharose Fast Flow cartridge (GE Healthcare). IGPD2 does not bind to the DEAE matrix under the conditions used, so the column was washed with buffer A and a column-volume of eluted material was retained. Protein was precipitated in 1 M ammonium sulphate solution, pelleted by centrifugation at 40000 g for 5 min and dissolved in buffer B (50 mM Tris-HCl pH 8.0, 0.8 M ammonium sulphate). Hydrophobic chromatography was carried out with a Phenyl-Toyopearl 650S (TOSOH) column or using a 5 ml Hi-Trap Phe-HP cartridge (GE Healthcare) that had been pre-equilibrated with buffer B. The sample was applied and IGPD2 was eluted by a reverse gradient of ammonium sulphate concentration from buffer B to buffer A. Fractions were collected and analysed for protein content by Bradford Assay (Bio-Rad). Peak protein fractions containing IGPD2 were combined and the protein was precipitated in 2 M ammonium sulphate solution. Precipitate was collected by centrifugation at 40000 g for 5 mins and dissolved in 1 ml of buffer C (50 mM Tris-HCl

pH 8.0 and 0.5 M NaCl). The sample was applied to a 16 x 60 Superose 6 gel filtration column pre-equilibrated in buffer C. Gel filtration was performed at a flow rate 1.5 ml min^{-1}. 2 ml fractions were collected and those with the highest protein concentration were combined. The final samples were concentrated with a 30000 MW cut-off VivaSpin concentrator and buffer exchanged into buffer D (50 mM Bis-Tris propane pH 8.0, 50 mM NaCl, 10 mM β-mercaptoethanol and 0.4 mM $MnCl_2$) using a diafiltration cup, then analysed by SDS-PAGE.

Crystallisation

Prior to crystallisation, both wild-type IGPD2 and the E21Q mutant were concentrated to 10 mg ml^{-1} in buffer D. Na_2HPO_4 or 1,2,4-triazole (Sigma) were added to the wild-type enzyme to a final concentration of 5 mM prior to crystallisation. The same procedure was followed for the E21Q mutant with synthetic IGP (Toronto Research). Initial conditions were determined by automated screening (Nextal) using a Matrix Hydra II crystallisation robot. The conditions were optimized by hanging-drop vapor diffusion using a 5:1 ratio of protein to precipitant, to yield cubic crystals after 5-10 days when equilibrated against a 1 ml reservoir of precipitant at a temperature of 290 K. Crystals of the 1,2,4-triazole complex were grown in optimized handing-drop experiments from 0.1 M Tris-HCl pH 9.0 and 13 % PEG 200. Crystals of the phosphate complex were grown in sitting-drop experiments from 0.1 M Bicine pH 9.0 and 10% PEG 6000. The E21Q mutant complexes with IGP were crystallized in hanging drop experiments with 25 % ethylene glycol (form A) or 0.1 M Hepes pH 7.5, 0.05 % PEG 2000 and 0.4 M succinic acid pH 7.0 (form B).

Data collection

The wild-type co-crystals with 1,2,4-triazole or phosphate were cryoprotected in their crystallisation buffer containing 25 % ethylene glycol. The form A E21Q IGPD2 crystals were cryoprotected with 30 % ethylene glycol and the form B crystals were cryoprotected in their crystallisation buffer containing 30 % ethylene glycol, prior to cooling in liquid nitrogen. All datasets were collected on the MX beamlines at the Diamond Light Source (Table 1). The two mutant complexes with IGP and the 1,2,4-triazole complex belonged to the spacegroup P432 with cell dimensions of approximately a≈113.5 Å and with a monomer in the asymmetric unit. The structure with phosphate was determined to be in the spacegroup I23 with cell dimensions of a=225 Å and eight subunits of IGPD2 in the asymmetric unit. Data collection statistics are summarized in Table 1.

Structure determination and Refinement

Data were processed at the Diamond Light Source using the Xia2 pipeline (Winter, 2010) or by integration using imosflm (Leslie and Powell, 2007). All additional data processing was carried out using CCP4i (Winn et al., 2011). The phosphate complex was determined by molecular replacement using PHASER (McCoy et al., 2007) with a monomer of *A. thaliana* IGPD1 (PDB: 2F1D) (Glynn et al., 2005) as a search model (sequence identity ~96%). The complex with 1,2,4-triazole was determined by the same method, but using a monomer of IGPD2 from the phosphate complex as the search model. All subsequent structures were determined directly by refinement using protein coordinates from the 1,2,4-triazole complex, but omitting ligands, metal ions and solvent. Model building and refinement was carried out in COOT (Emsley and Cowtan, 2004) and Refmac5 (Murshudov et al., 1997), with ligand libraries generated by JLigand (Lebedev et al., 2012). Refinement statistics are summarized in Table 1.

Models were validated using Molprobity (Chen et al., 2010) and figure were made in Pymol (Schrodinger, 2010). Refinement statistics are summarized in Table 1.

In the case of the form A E21Q complex with substrate, the very high resolution (1.12 Å) of the data meant that there was sufficient evidence to interpret the maps as corresponding to the binding of both the 2*R*3*S* (substrate) and 2*S*3*S* (contaminant) diastereoisomers of IGP (Figure S3). Initial maps showed that the two ligands share a common position for the phosphate moiety and the C1 and C2 atoms, but since the diastereoisomers differ in chirality at C2, two positions could be observed in the electron density map for the C2-OH. The substrate and 2*S*3*S* IGP share the same chirality at C3, consistent with the observation of a single electron density feature representing the C3-OH, which is a ligand to Mn1. The structure was refined cautiously with each of the diastereoisomers of IGP first modeled on their own into the corresponding density. Difference maps subsequently revealed the unmodelled position for the binding of the alternate diastereoisomer. Only one of the ring positions was compatible with the chirality of the substrate, due to a steric clash with the C2-OH, resulting in the substrate and 2*S*3*S* IGP being assigned the neutral imidazole and an imidazolate, respectively. Unlike the electron density at Mn1, which was spherical, that at Mn2 was ellipsoidal, suggesting two different metal positions, each associated with the binding of the different diastereoisomers.

Determining the Km of IGP binding to IGPD2

The Km was determined using a modified coupled assay (Hawkes et al., 1995). Assay buffer (0.1 M potassium-Hepes pH 7.0, 0.22 M Ammonium chloride and 0.22 M L-glutamate sodium salt) was aliquoted into 10 ml volumes and frozen at -80 ^{o}C. Prior to use the assay buffer was defrosted and supplemented with 0.2 mM $MnCl_2$ and 0.01

mM pyridoxal-5-phosphate, both of which were made up in 0.1 M potassium-Hepes pH 7.0. Stock solutions of NADH and bovine glutamate dehydrogenase (GDH) (both Sigma) were prepared in 0.1 M potassium-Hepes pH 7.0 to 6 mM and 2 mg ml^{-1}, respectively. Imidazoleacetol-phosphate transaminase (IAPT), which had been previously purified and frozen as droplets (S. Singh, Syngenta), was defrosted on ice. IGP, supplied as a salt (Toronto Research), was made up to 0.2 M in potassium-Hepes pH 7.0. Mn-IGPD2 from frozen stocks was diluted 40-fold in assay buffer to a working concentration of 4.2 µM. 650 µl of supplemented assay buffer was combined with 100 µl of GDH, 100 µl of IAPT (large excess) and 50 µl of NADH in a cuvette and incubated at 30 ^{o}C in a temperature-controlled spectrophotometer. 10 µl of IGPD2 was added and the signal was recorded at 340 nm until it had stabilized at which point 10 µl of IGP at various concentrations (125 µM to 2 mM) was added to start the reaction. After a lag time of ~120 s, initial rates from the linear part of the curve were recorded. Km and Kcat were determined by non-linear least squares fitting in Graph Pad Prism.

Cloning, expression and purification of the ΔC mutant of Pf IGPD

The gene encoding IGPD from *Pyrococcus furiosus* was PCR amplified using the synthetic oligonucleotides *Pf*-IGPD_f and *Pf*-IGPD_r. These were designed to incorporate a 5' mutation to replace the start codon GTG, commonly observed in Archea, with an ATG, prior to cloning into pETBlue (Novagen). The subsequent plasmid, pIGPDKLB, was transformed into Tuner (DE3) *E. coli* (Novagen) and *Pf* IGPD was over-expressed at 37 °C for 3 hr post-addition of 1 mM IPTG and 4 mM $MnCl_2$. A second construct was amplified from pIGPDKLB using a pair of synthetic oligonucleotide primers, *Pf*-IGPD-ΔC_f and *Pf*-IGPD-ΔC_r, to introduce a premature stop codon into the 3' end of the gene encoding *Pf* IGPD, thereby removing the part of

the sequence that encodes for the C-loop. The amplified gene was subsequently cloned into a pET24a vector, producing the plasmid pΔC-IGPDKLB, which was over-expressed using the same method as the original construct. The wild-type protein was purified using an initial 20 min heat step at 70 ^{o}C in buffer E (40 mM Tris pH 8.0 and 2 mM EDTA), followed by centrifugation to pellet the cell debris. The supernatant was applied onto a DEAE-sepherose fast flow column (Amersham Biosciences) and eluted on a gradient of 0-0.5 M NaCl in buffer E. The wild-type protein was then concentrated and applied to a Hi-Load Superdex 200 gel filtration column, eluting as the 24mer (~500 kDa) species. Purification of the ΔC mutant followed the same method as the wild-type protein except that the ΔC mutant was precipitated by addition of 2 M ammonium sulphate and pelleted by centrifugation at 40000g for 5 min, before being resuspended in buffer F (40 mM Tris pH 8.0, 2 mM EDTA and 0.5 M NaCl) prior to gel filtration. The ΔC mutant eluted as the trimeric (~60kDa) species, which was reapplied to the gel filtration column in the presence of 2 mM $MnCl_2$ in order to reassemble it. Both proteins were concentrated using a 30000 MW cut-off VivaSpin concentrator and buffer exchanged into 0.1 M Hepes pH 7.0 using a diafiltration cup.

Supplemental References:

Chen, V.B., Arendall, W.B., III, Headd, J.J., Keedy, D.A., Immormino, R.M., Kapral, G.J., Murray, L.W., Richardson, J.S., and Richardson, D.C. (2010). MolProbity: all-atom structure validation for macromolecular crystallography. Acta Crystallogr D *66*, 12-21.

Emsley, P., and Cowtan, K. (2004). Coot: model-building tools for molecular graphics. Acta Crystallogr D *60*, 2126-2132.

Glynn, S.E., Baker, P.J., Sedelnikova, S.E., Davies, C.L., Eadsforth, T.C., Levy, C.W., Rodgers, H.F., Blackburn, G.M., Hawkes, T.R., Viner, R., *et al.* (2005). Structure and mechanism of imidazoleglycerol-phosphate dehydratase. Structure *13*, 1809-1817.

Hawkes, T.R., Thomas, P.G., Edwards, L.S., Rayner, S.J., Wilkinson, K.W., and Rice, D.W. (1995). Purification and Characterization of the Imidazoleglycerol-Phosphate Dehydratase of Saccharomyces-Cerevisiae from Recombinant Escherichia-Coli. Biochem J *306*, 385-397.

Kleywegt, G.J., Zou, J.Y., Kjeldgaard, M., and Jones, T.A. (2001). International Tables for Crystallography, Vol F.

Lebedev, A.A., Young, P., Isupov, M.N., Moroz, O.V., Vagin, A.A., and Murshudov, G.N. (2012). JLigand: a graphical tool for the CCP4 template-restraint library. Acta Crystallogr D *68*, 431-440.

Leslie, A.G.W., and Powell, H.R. (2007). Processing diffraction data with MOSFLM, in Evolving Methods for Macromolecular Crystallography, Nato Science Series *245*, 41-51.

McCoy, A.J., Grosse-Kunstleve, R.W., Adams, P.D., Winn, M.D., Storoni, L.C., and Read, R.J. (2007). Phaser crystallographic software. J Appl Crystallogr *40*, 658-674.

Murshudov, G.N., Vagin, A.A., and Dodson, E.J. (1997). Refinement of macromolecular structures by the maximum-likelihood method. Acta crystallographica Section D, Biological crystallography *53*, 240-255.

Schrodinger, LLC (2010). The PyMOL Molecular Graphics System, Version 1.3r1.

Winn, M.D., Ballard, C.C., Cowtan, K.D., Dodson, E.J., Emsley, P., Evans, P.R., Keegan, R.M., Krissinel, E.B., Leslie, A.G., McCoy, A., *et al.* (2011). Overview of the CCP4 suite and current developments. Acta Crystallogr Sect D *67*, 235-242.

Winter, G. (2010). xia2: an expert system for macromolecular crystallography data reduction. J Appl Crystallogr *43*, 186-190.
