# Supplementary figures and images for: Crystal Structures Reveal that the Reaction Mechanism of Imidazoleglycerol-Phosphate Dehydratase Is Controlled by Switching Mn(II) Coordination
Source: Structure. 2015 Jul 7;23(7):1236–45. doi: 10.1016/j.str.2015.05.012 (PMC4509728; doi:10.1016/j.str.2015.05.012)

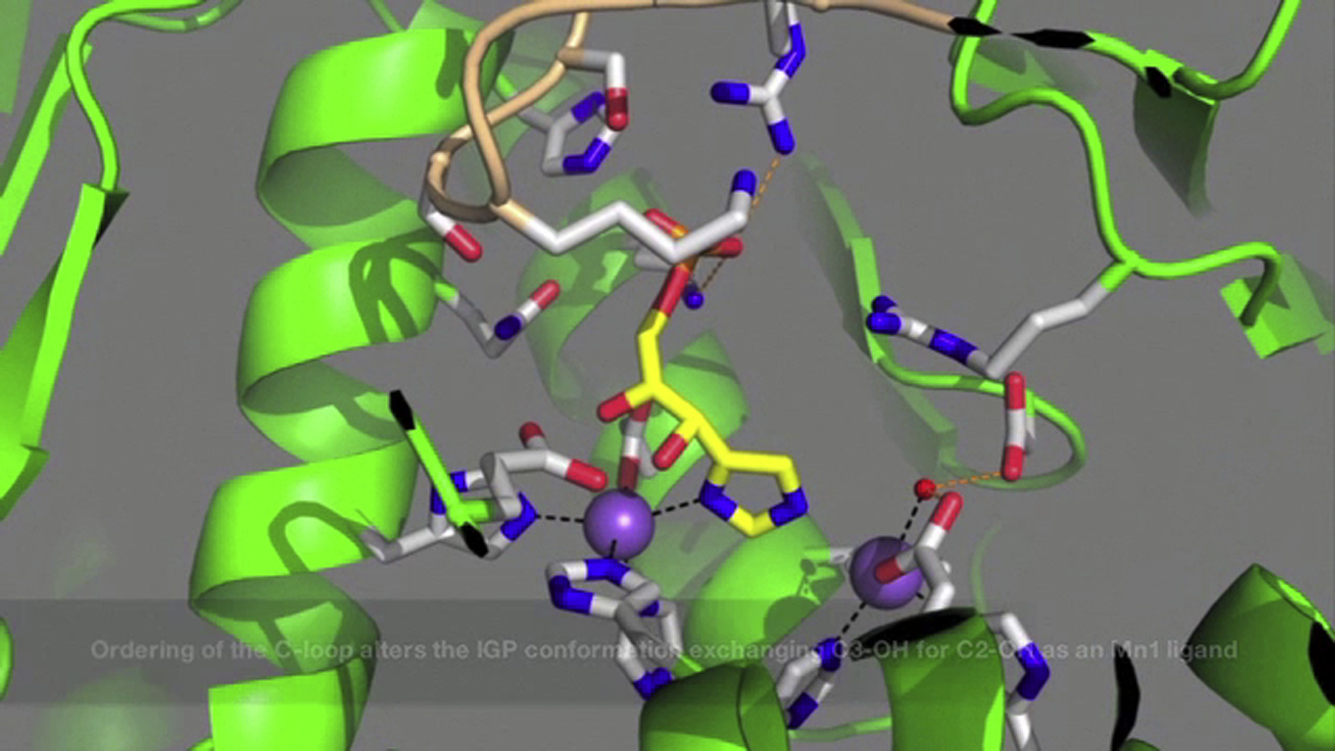

Supplement: Movie S1. The Reaction Mechanism of IGPD [file mmc2.jpg]
